# The Effects of Strain Rates on Mechanical Properties and Failure Behavior of Long Glass Fiber Reinforced Thermoplastic Composites

**DOI:** 10.3390/polym11122019

**Published:** 2019-12-05

**Authors:** Junjia Cui, Shaoluo Wang, Shuhao Wang, Guangyao Li, Peilin Wang, Chengsong Liang

**Affiliations:** 1 State Key Laboratory of Advanced Design and Manufacturing for Vehicle Body, Hunan University, Changsha 410082, China; cuijunjia@hnu.edu.cn (J.C.); wsl0809@hnu.edu.cn (S.W.);; 2 Capital Aerospace Engineering Machinery Company, Beijing 100076, China

**Keywords:** long glass fiber reinforced polypropylene composites (LGFRPPs), high strain rates, mechanical properties, failure mechanism

## Abstract

Long glass fiber reinforced thermoplastic composites have been increasingly used in automotive parts due to their excellent mechanical properties and recyclability. However, the effects of strain rates on the mechanical properties and failure mechanisms of long glass fiber reinforced polypropylene composites (LGFRPPs) have not been studied systematically. In this study, the effects of strain rates (from 0.001 s^−1^ to 400 s^−1^) on the mechanical properties and failure mechanism of LGFRPPs were investigated. The results showed that ultimate strength and fracture strain of the LGFRPPs increased obviously, whereas the stiffness remained essentially unchanged with the strain rates from low to high. The micro-failure modes mainly consisted of fibers pulled out, fiber breakage, interfacial debonding, matrix cracking, and ductile to brittle (ductile pulling of fibrils/micro-fibrils) fracture behavior of the matrix. As the strain rates increased, the interfacial bonding properties of LGFRPPs increased, resulting in a gradual increase of fiber breakage at the fracture surface of the specimen and the gradual decrease of pull-out. In this process, more failure energy was absorbed, thus, the ultimate strength and fracture strain of LGFRPPs were improved.

## 1. Introduction

Glass fiber reinforced thermoplastic (GFRT) composites have a widespread utilization in automotive components due to their advantages of light weight, high specific strength, design flexibility, recyclability, etc. [1,2,3,4,5,6]. The GFRT composite can be divided into short (average fiber length of less than 1 mm), long (average fiber length in the 1–25 mm range), and continuous (average fiber length equal to the part dimension) types of GFRT composite [7,8,9,10]. Recently, scholars have been willing to pay attention to GFRT composites with respect to their failure behaviors and ability to resist loads, especially in the car collision.

The effects of strain rates on mechanical properties of short GFRT (SGFRT) composite were investigated in several works [11,12,13,14,15]. The previous results indicated that the composite was a strain rate-dependent material, and its tensile strength and stiffness increased with the increasing of the strain rates. Additionally, the effects of fiber content, strain rate, and weld-line on tensile strength, modulus, and fracture toughness of SGFRT composites with 0–40% of fiber weight content were also studied [16,17,18]. It was found that tensile strength, modulus, and fracture toughness of the composite increased with the increasing volume fraction of the fibers and the natural logarithm of the strain rates.

The effects of strain rates on the mechanical behavior of continuous GFRT composites [19,20,21,22] have drawn attention from many scholars in the past years. The strain rate-dependent tensile behavior of continuous GFRT composite under off-axis compression [19] and tensile [20] loading was studied. The experimental results showed that the mechanical properties were apparently dependent on strain rates. Brown et al. [21] reported the effects of strain rates on the tensile, shear, and compression behavior of continuous GFRT composites over a strain rate range of 10^−3^ s^−1^–10^2^ s^−1^. They observed that the tensile and compression modulus and strength increased with the increase in the strain rates. However, the shear modulus and strength were discovered to decrease with the increasing strain rates, and the strain rate effects in the material appeared to be influenced primarily by the matrix viscoelasticity, fiber-matrix interfacial properties, the composite woven reinforcement architecture, and the time-dependent nature of damage accumulation. Duan et al. [22] discussed the effects of strain rates on the tensile properties of hot-molded continuous GFRT composite. The results showed that the composite was sensitive to strain rates, and its ultimate strain and ultimate strength increased with increasing strain rates.

Mechanical properties of GFRT composites increase with the fiber length while its processability decreases [9]. Compared with SGFRT composites, long GFRT composites have better mechanical properties and wider applications. Meanwhile, by comparison with continuous GFRT composites, long GFRT composites have the advantages of short production cycle, good processability, low cost, and ease of realizing mass production. The effects of impact loading on tensile properties and microscopic failure mechanism of long glass fiber reinforced polypropylene composites (LGFRPPs) are also studied. Zhang et al. [23] successfully established a two-phase homogenized material model of LGFRPPs under low velocity impact loading and presented a method to evaluate the energy absorption capability. They found that the material behavior was anisotropic and strain rate-dependent. Properties of LGFRPPs subjected to transverse intermediate velocity blunt object impact were explored by Bartus et al. [24]. The results showed that the material was not sensitive to the loading rate, but it had high impact energy dissipation. The predominant energy dissipation mechanisms were fiber fracture, fiber debonding, fiber pull-out, and matrix fracture. An investigation of the strain rate-dependent multiaxial characterization of LGFRPPs was performed [25] using tensile-, notched tensile-, shear tensile-, and punch tests in a range of strain rates of about 10^−3^ s^−1^ up to 10^2^ s^−1^. It was found that the adiabatic heating enlarged the deformation zone, impeded strain localization, and led to higher energy absorption at higher strain rates. The strain rate dependencies differed with the fiber orientation. The matrix around the fibers was highly deformed due to the increase of the interface cohesion at higher strain rates.

The studies mentioned above mainly focus on the effects of strain rates on mechanical properties of SGFRT composite or continuous GFRT composite. The effects of high strain rates on the mechanical behavior of LGFRPPs with fiber lengths of about 1–25 mm have not been investigated systematically.

In this paper, LGFRPPs pellets were manufactured by melt impregnation technology (continuous melt-coating method), and specimens were fabricated by the injection molding method [7,26,27]. The strain rate-dependent static and dynamic mechanical properties of LGFRPPs were investigated at various strain rates from 0.001 s^−1^ to 400 s^−1^. High-speed camera and digital image correlation (DIC) systems were used to analyze the tensile behavior and full-field strain of the LGFRPPs. The fracture behavior and failure mechanism were characterized by scanning electron microscope (SEM).

## 2. Materials and Methods

### 2.1. Materials

The LGFRPPs pellets (with 40% of fiber weight content) used in this study were composed of polypropylene and E-glass fiber. Polypropylene (PP F401) was supplied by Sinopec Yangzi Petrochemical Co., Ltd. (Nanjing, China). The E-glass fibers and long pellets were provided by Kunshan SOFA Plastic Chemical Co., Ltd. (Suzhou, China). The properties of the fiber, PP, and composites are listed in Table 1. Wherein the length of glass fiber was approximately 12 mm and diameter was 0.013 mm, the aspect ratio was 923.

### 2.2. Specimen Preparation

**Figure 1:**
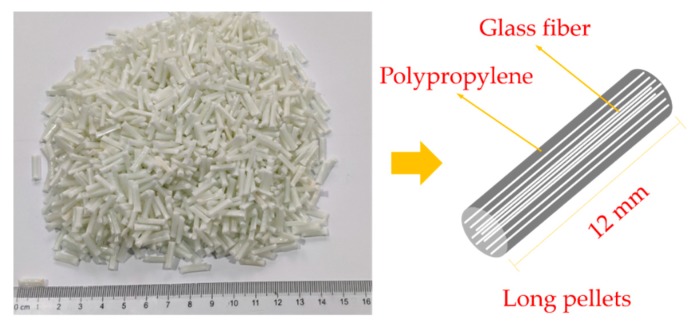
Long pellets and model diagram.

**Figure 2:**
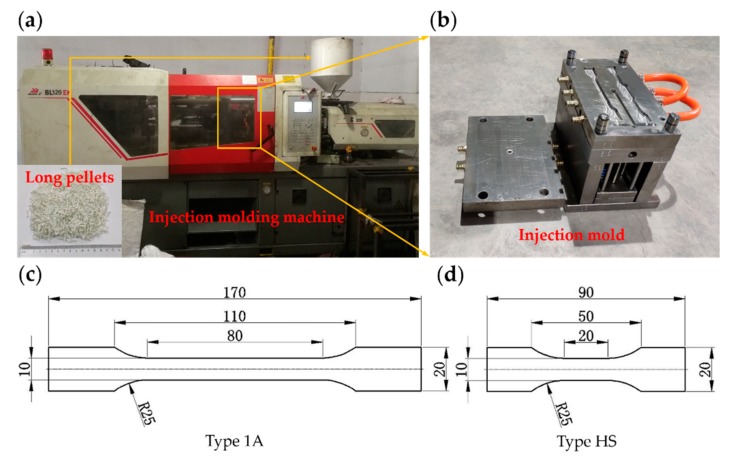
Sample preparation process: (**a**) Injection molding machine, (**b**) injection mold, (**c**) specimen shape and dimensions of Type 1A, and (**d**) specimen geometry of Type HS.

Experimental specimens were prepared by injection molding method. Firstly, the LGFRPP pellets were fabricated by mixing E-glass fibers and the PP matrix with melt cladding method in a twin-screw extruder. That is, the preheated glass fiber entered the cross cladding die through the guide wheel, and the continuous glass fibers were coated by the PP melt in the die, and then the required long pellets were obtained by cooling and cutting [7,26,27]. The long pellets and model diagram are shown in Figure 1. Secondly, the long pellets were dried for 48 h before injection molding. Finally, the long pellets were melted in the injection molding machine by heating to around 200 °C, and the LGFRPPs samples were made by pressurizing, injecting, cooling, and disengaging. The clamping force of the injection molding machine (BOLE BL120EK, Ningbo, China) was 120 tons. The injection molding machine and injection mold are shown in Figure 2a,b. The dumbbell specimens were cut along the injection flow direction (IFD) from the injection-molded LGFRPP samples. The dimensions of the static tensile specimen (named Type 1A) were injection molded according to the ISO 527-2 norm. Since there is not yet a valid standard for the execution of the high-speed tensile tests for polymers or polymer matrix composites, the dimensions of the dynamic tensile specimen (named Type HS) were designed with reference to the ISO 26203-2 and ISO 527-2 norms. The Type HS specimen size was to achieve higher strain rates with the testing machine [1,22,25]. Both of the specimen thicknesses were 4 mm. The shapes and dimensions of the specimens in millimeters are shown in Figure 2c,d.

### 2.3. Tensile Testing Methods

**Figure 3:**
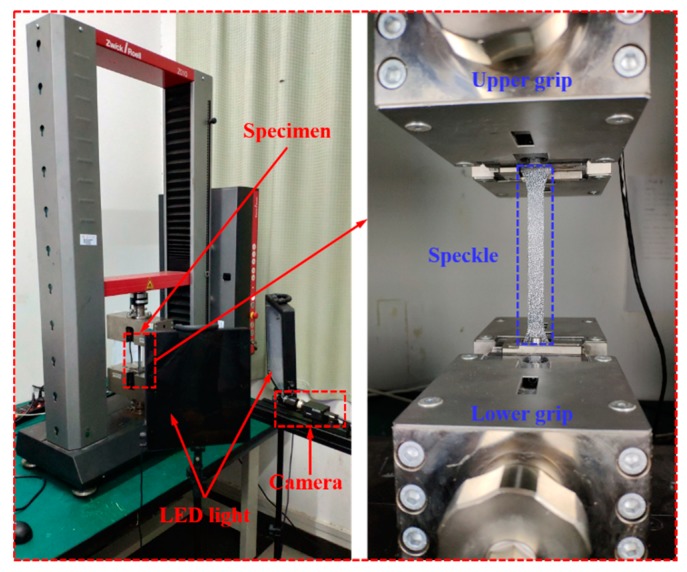
The devices for static tensile tests.

**Figure 4:**
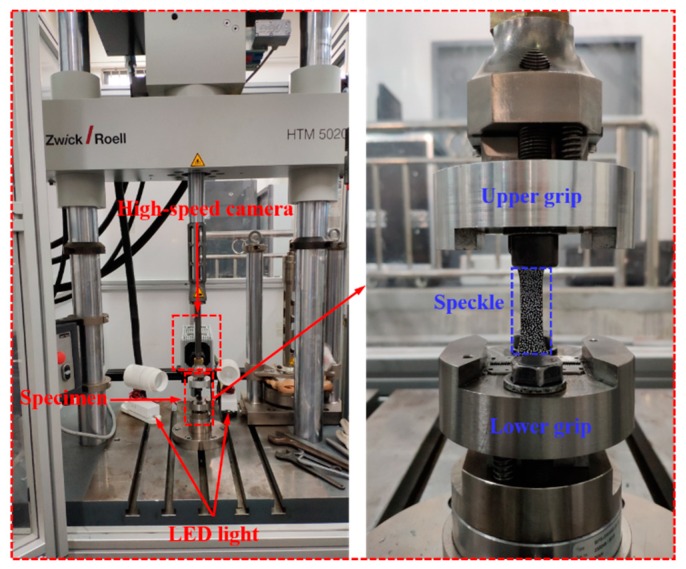
The devices for high-speed tensile tests.

The static tensile tests were implemented using a Zwick/Roell Z010 universal materials testing machine (Ulm, Germany) with a maximum load of 10 kN, as shown in Figure 3. The tests were carried out at the strain rates of 0.001 and 0.01 s^−1^. High-speed tensile tests were performed on a Zwick/Roell HTM5020 high-speed testing system (Ulm, Germany) with a maximum test velocity of 20 m/s, as well as a maximum load of 50 kN, as shown in Figure 4. The dynamic tensile tests were carried out at the strain rates of 1, 10, 100, 200, and 400 s^−1^.

The tensile tests with the same loading conditions were repeated three times. In order to obtain full-field strain contours, strain evaluation was performed for all groups of tensile tests by the aid of the DIC system. DIC was an optical method to track changes in a series of deformed images. It was used to measure displacements and strains on the surface of a deforming material in a non-contact way [28]. The DIC device used in this paper was produced by Correlated Solutions Inc., America (Irmo, SC, USA). The distance between the aperture of the lens and the test specimen (stand-off distance, SOD) of the low-speed test system was determined as 700 mm, while the SOD of the dynamic test system was determined as 400 mm. The resolution of length per pixel was 0.125 mm/pixel. The resolution of area-of-interest (AOI) for the static tests was 640 × 80 pixels and the resolution of AOI for the dynamic tests was 160 × 80 pixels. The specimen Type 1A images were recorded by a low-speed camera (Grasshopper3 GS3-U3-60S6M CCD) with the resolution of 2736 × 2192 pixels. The low-speed camera was made by Point Grey Research, Inc., Richmond, BC, Canada. And the frame rate was set as 7.5 frames per second. In addition, the specimen Type HS images were recorded by a high-speed camera (FASTCAM SA-X2) with a maximum resolution of 1024 × 1024 pixels. The high-speed camera (FASTCAM SA-X2) was made by Photron Limited, Tokyo, Japan. The sampling rate of the high-speed camera was set to be 5000, 50,000, 90,000, 120,000, and 135,000 frames per second, respectively. This method had been proved to be an efficient strain recording method for composites [22,29,30,31,32,33]. The post-processing analysis of all the measurement results was conducted with the DIC professional software (VIC 2D 6, Correlated Solutions Inc., Irmo, SC, USA).

### 2.4. Microscopic Characterization

An FEI Quanta 200 scanning electron microscope (FEI Company, Hillsboro, OR, USA) was used to analyze the failure mechanism and interface characteristics of LGFRPPs fracture surface.

## 3. Results and Discussion

### 3.1. Full-Field Strain Analysis

The tensile experiments were implemented under uniaxial tensile loading, hence, only the axial strain was calculated and analyzed. Simultaneously, all the strain values were engineering strain values obtained by VIC 2D 6 processing. The parallel length of the tensile specimen was selected to be the calculation area. The calculation area was divided into a large number of calculation subsets, where the size of the calculation subset was arranged as 21 × 21 pixels. The corresponding step size was 5. The strain was calculated result from the average displacement of a couple of adjacent calculation subsets, so the strain at the edge could not be determined [29,34,35,36,37,38].

**Figure 5:**
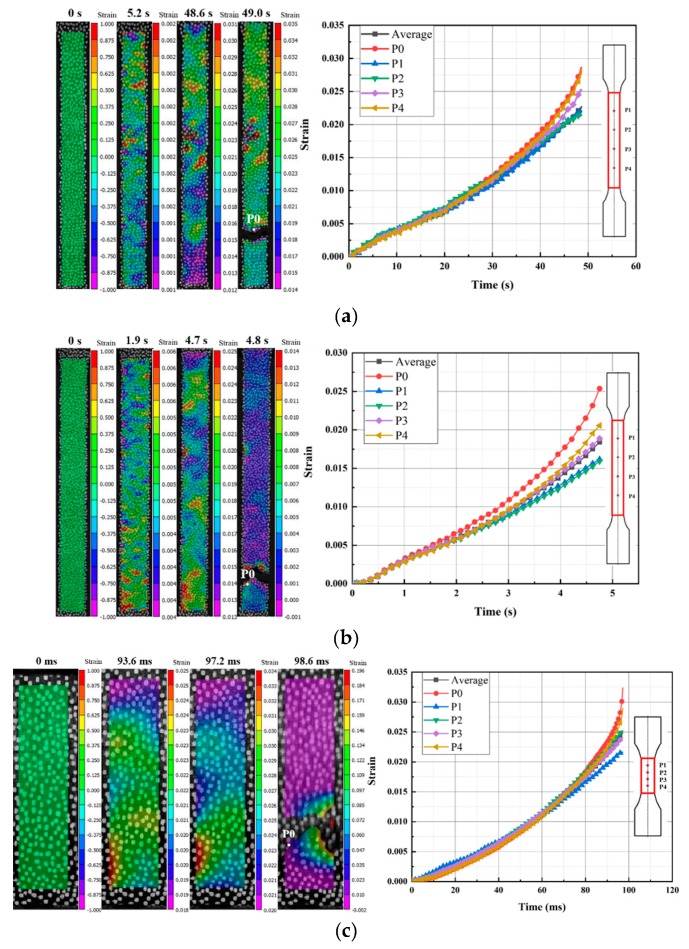

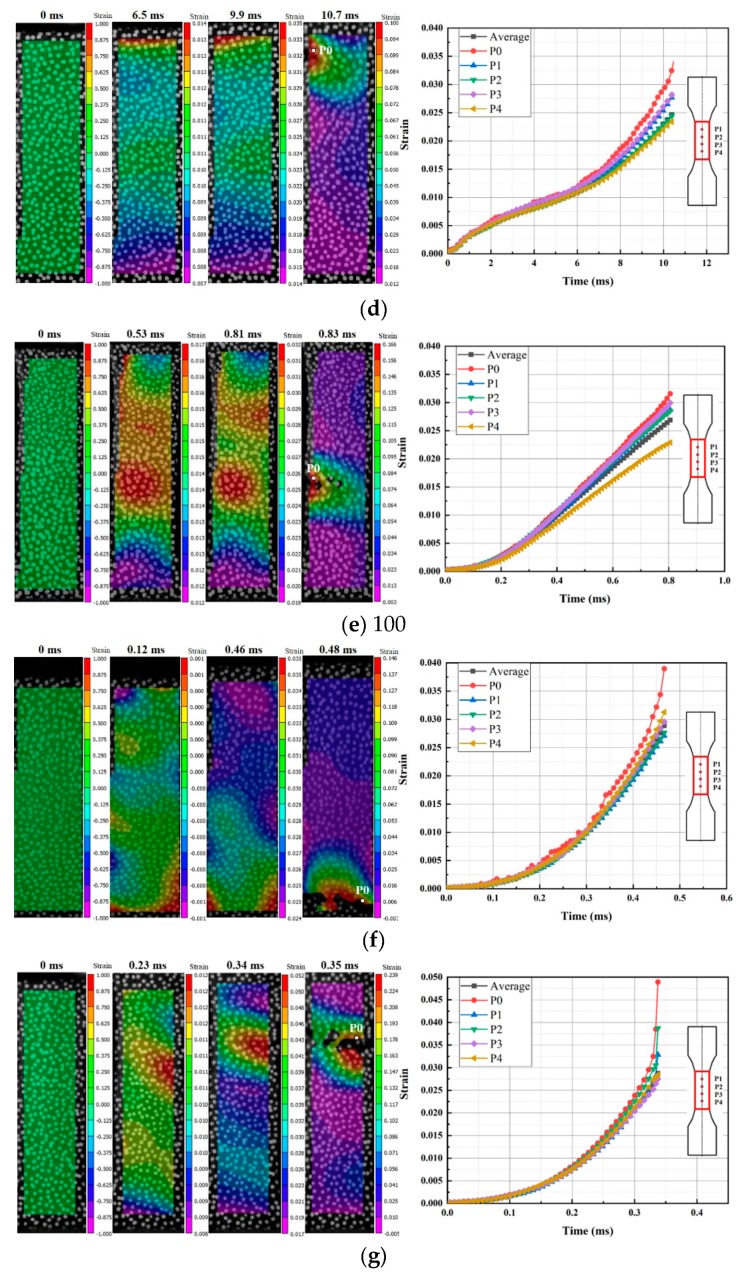
The engineering strain contours and engineering strain-time curves of progressive failure process of the tensile specimens at different strain rates: (**a**) 0.001 s^−1^, (**b**) 0.01 s^−1^, (**c**) 1 s^−1^, (**d**) 10 s^−1^, (**e**) 100 s^−1^, (**f**) 200 s^−1^, and (**g**) 400 s^−1^.

The strain-time curves of some special points distributed on the specimen surface and the average strain-time curve of the calculation area was extracted. P_0_ was the point near the fracture crack. P_1_, P_2_, P_3_, and P_4_ were four reference points distributed on the tensile axis of the specimen from top side to bottom side. The distances between two adjacent reference points at static and dynamic strain rates were 16 mm and 4 mm, respectively. The engineering strain contours and the engineering strain-time curves are shown in Figure 5. Meanwhile, full-field strain analysis results of the tensile process are listed in Table 2. For the selected seven strain rates, the fracture time of the specimens was 49.0 s, 4.8 s, 98.6 ms, 10.7 ms, 0.83 ms, 0.48 ms, and 0.35 ms, respectively. This showed that the fracture time decreased with the increase of tensile velocity, and the ratio of the fracture time of two strain rates was approximately equal to the inverse ratio of the corresponding strain rate. According to the full-field strain analysis results, with the increase of strain rate from 0.001 s^−1^ to 400 s^−1^, the ultimate average strain of the parallel length region increased from 0.018 to 0.030, an increase of 67%, and the fracture strain (P_0_) increased from 0.027 to 0.047, an increase of 74%. Therefore, LGFRPPs under tensile loading were characterized by strain rate effects.

The deformation mainly concentrated near the fracture crack or the location of possible failure, while the strain far from the fracture crack or the location of possible failure changed a little. It could be seen that the fracture strain of P_0_ was larger than the ultimate average strain of the parallel length region at the same strain rate in Figure 5. In addition, the deviation between them was becoming more obvious due to the strain localization with the tensile processed [34].

### 3.2. Effect of Strain Rate on the Mechanical Behaviors

**Figure 6:**
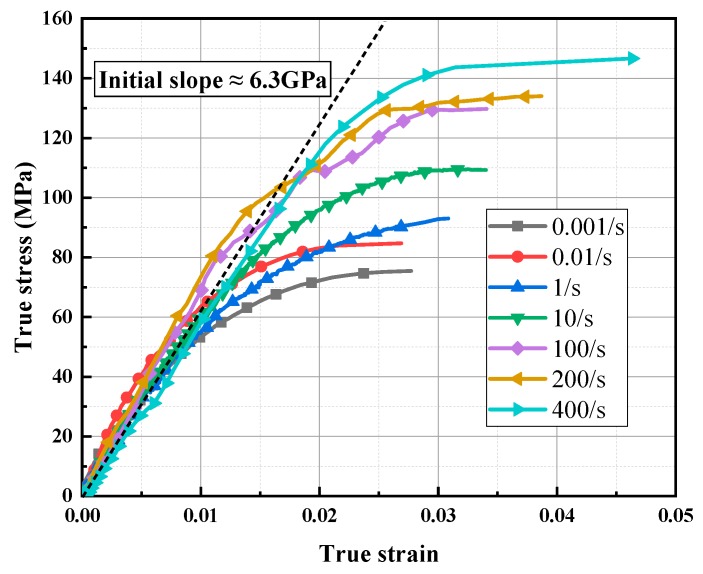
True stress-strain curves for LGFRPPs at different strain rates: 0.001–400 s^−1^.

The specimen strains were determined using DIC and the evaluated strain values were obtained from the fracture strain of P_0_ [25,34]. According to the true stress-strain curves of LGFRPPs, as presented in Figure 6, the LGFRPPs were strongly sensitive to the strain rates. The stress-strain curves at strain rates from 0.001 s^−1^ to 0.01 s^−1^ (low strain rates) could be divided into two stages: elastic deformation and plastic hardening, then directly fracture. The stress-strain curves at the strain rates of 1 s^−1^ and 10 s^−1^ (medium strain rates) presented a straight rising process till peak stress followed by abrupt failures, and the tendency was similar to that of the hot-molded continuous GFRT composite (in case of strain rates 0.001 s^−1^–50 s^−1^, the curves showed more straight rising process until peak stress followed abrupt failures) [22]. The stress-strain curves at strain rates of 100, 200, and 400 s^−1^ (high strain rates) showed totally different characteristics in comparison with lower strain rate curves. These three curves had significant fluctuation phenomena before maximum stress, followed by abrupt failures. The fluctuation phenomena of the curves might be related to damage accumulation. Since the interfacial debonding was the main micro-damage mode before specimen failure, the change of stress-strain response was mainly caused by strain rate sensitive failure behavior of interface properties which was attributed to the strain rate sensitivity of the fiber and matrix [39,40].

**Figure 7:**
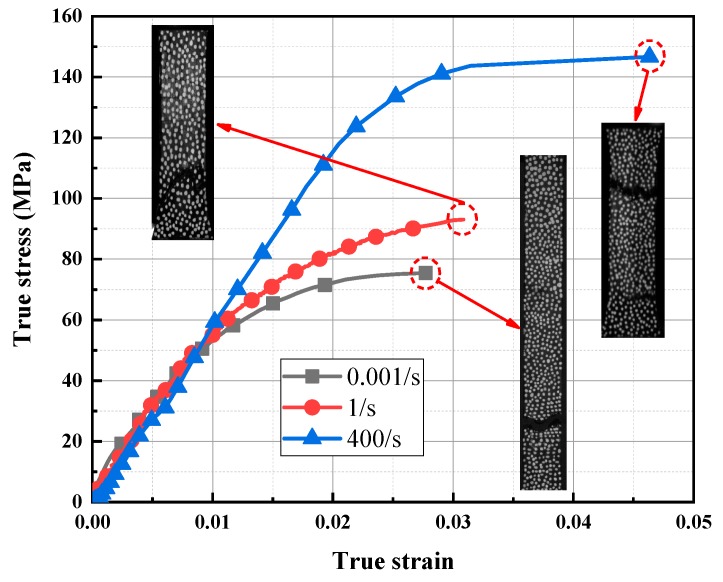
Typical true stress-strain curves and tensile failure morphology at low, medium, and high strain rates.

The true stress-strain curves of 0.001 s^−1^, 1 s^−1^ and 400 s^−1^ were selected as the typical low, medium, and high strain rate analysis curves, as shown in Figure 7. It could be found that the ultimate strength and fracture strain of the stress-strain curve increased obviously, whereas the modulus (initial elastic slope) remained essentially unchanged with the strain rates from low to high. The energy absorption capacity also improved significantly at the higher strain rates. Observing the curves and fracture morphology, it was found that the LGFRPPs presented obviously brittle fracture behavior. This might be due to the low ductility of the glass fiber. Meanwhile, the fluctuating instability of the stress-strain curve might be caused by microscopic defects.

The elasticity modulus, ultimate strength, and total elongation of tensile loading tests at various strain rates are summarized in Table 3. When the strain rates increased from 0.001 s^−1^ to 400 s^−1^, the ultimate tensile strength increased from 75.4 MPa to 146.7 MPa, which was improved almost two times, while the total elongation increased from 2.8% to 4.6%, which was also improved nearly twice. The elastic modulus fluctuated at 6.3 GPa, and the fluctuation range was from 5.2 GPa to 7.3 GPa, which remained essentially constant with the ascending strain rates. Thus, the strain rates extremely affected the tensile strength and deformation. However, the stiffness might be insensitive to the strain rates.

**Figure 8:**
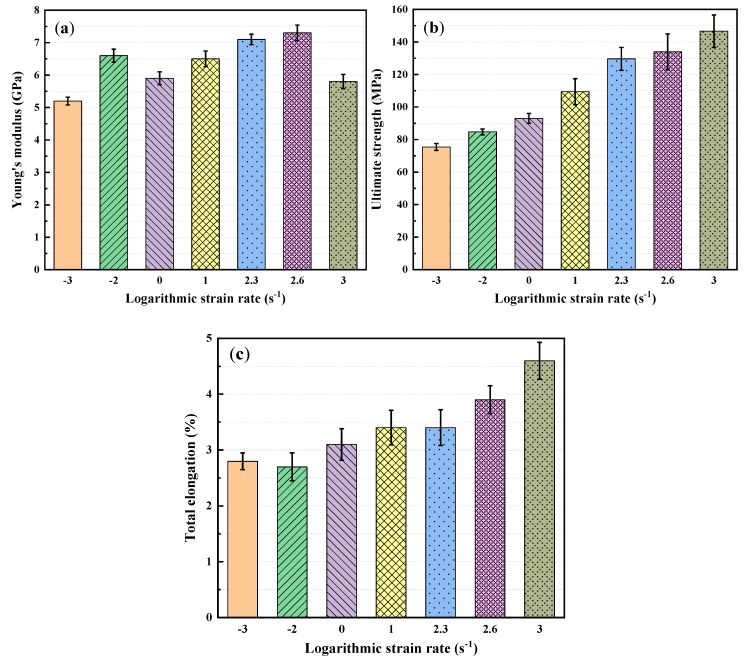
Effects of logarithmic strain rates on mechanical properties of LGFRPPs: (**a**) Young’s modulus, (**b**) ultimate strength, and (**c**) total elongation.

The effects of logarithmic strain rates on the mechanical properties of LGFRPPs are shown in Figure 8. The variation law is consistent with the strain rate effects mentioned above. The effects of the strain rates on the elastic modulus, ultimate strengths, and total elongations might be caused by various factors or their coupling including viscous behavior of matrix, properties and orientation distribution of the fiber, interface properties between the matrix and fiber, and defects in the preparation process, etc. Furthermore, the strain rate sensitivity of the tensile strength could also be attributed to the strain rate dependence of the glass fibers in the fiber-dominated loading mode [21,22]. Therefore, long glass fibers played a dominant role in the injection-molded polymer matrix composite under tensile loading.

### 3.3. Microscopic Failure Modes and Mechanisms Analysis

#### 3.3.1. Micro-Failure Modes

**Figure 9:**
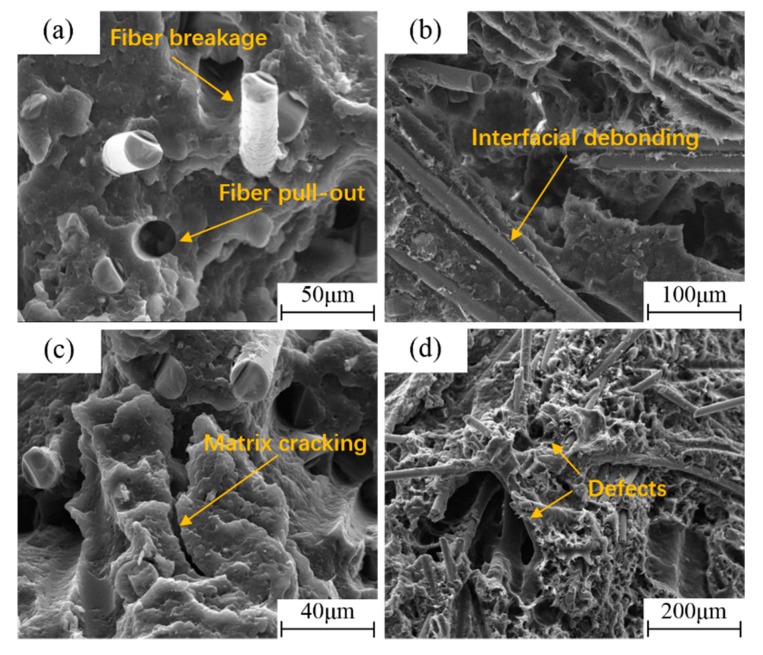
The morphology of typical fracture surface for LGFRPPs: (**a**) Fiber breakage and fiber pull-out, (**b**) interfacial debonding, (**c**) matrix cracking, and (**d**) defects.

The typical SEM fracture morphology of the LGFRPPs is shown in Figure 9. It indicates that the possible basic micro-failure modes involving matrix cracking, interfacial debonding, fiber breakage, and fiber pull-out could be observed on the specimen fracture surfaces. Figure 9a shows that the main micro-fracture modes of the LGFRPPs were fiber breakage and fiber pull-out. The interfacial debonding occurred at the early phase of LGFRPPs damage as shown in Figure 9b, which could explain the appearance of fibers breakage and fibers pull-out as the specimens broken. In Figure 9c, it could be seen that the crack in the fracture region had extended to the interface between the fiber and matrix, which resulted in fiber debonding and stress concentration.

At the same time, some larger bubbles and shrinkage cavities could be observed in matrix, as shown in Figure 9d, which might due to the defects in the manufacturing process of the specimens. These defects would cause stress concentration and lead to premature failure of the specimens. Actually, in the preparation of the experimental samples, after the long pellets were melted in the injection molding machine by heating, the samples were manufactured by pressurizing, injecting, cooling, and disengaging. Then the prepared samples were cut into specimens for tensile loading tests. In this process, some defects, such as bubbles, shrinkage cavities, flash, or warpage deformation, might emerge, which could be measured by nondestructive testing technology such as radiographic inspection techniques, ultrasonic testing, and acoustic emission testing technology. In order to reduce the amount of defects during the injection molding process, it was necessary to improve the fluidity of the matrix and the dispersal uniformity of fibers, and optimize the injection molding parameters, such as temperature, time, and pressure, etc. [22]. These defects might have a great influence on the mechanical behavior of LGFRPPs. Hence, the PP matrix with bubbles, shrinkage cavities and other defects would fracture firstly. When the load exceeded the critical load of crack propagation, the local defects would converge and cracks would propagate simultaneously. Therefore, the microscopic damage would be generated, including fiber breakage, fiber pull-out, interfacial debonding, and matrix cracking.

**Figure 10:**
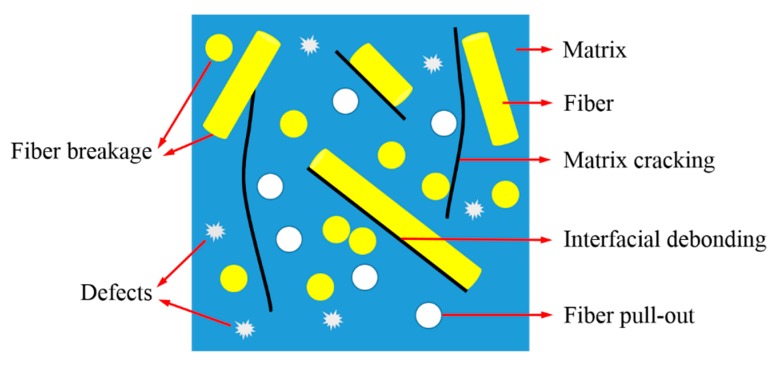
Schematic diagram of the microscopic failure modes.

A schematic diagram of the microscopic failure modes of LGFRPPs is shown in Figure 10. The main micro-failure modes could be summarized as: matrix cracking, interfacial debonding, fiber breakage, and fiber pull-out.

#### 3.3.2. Failure Mechanism Analysis

**Figure 11:**
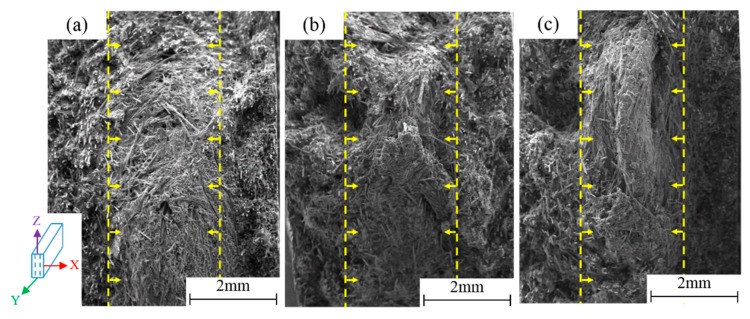
Fiber layered orientation distribution (sandwich structure) of the fracture surface morphology at different strain rates: (**a**) 0.001 s^−1^, (**b**) 1 s^−1^, and (**c**) 400 s^−1^.

The phenomenon of layered orientation distribution (sandwich structure) of the fibers was discovered along the specimen thickness direction (X-direction) at low, medium, and high strain rates. From Figure 11, the two thinner outer surface layers showed most fibers in the Y-direction (out of plane, equal to IFD), while the thicker core layer showed most fibers in the Z-direction, orthogonal to the IFD, showing a spiral orientation structure. It could be found that the stratification was consistent under different strain rates, which indicated that the layered structure was completely caused by the injection molding process and was independent of the strain rates. The fiber orientation distributions due to the injection molding process determined the anisotropy of the material. The sandwich structure might have a certain influence on the mechanical behavior of LGFRPPs [25].

**Figure 12:**
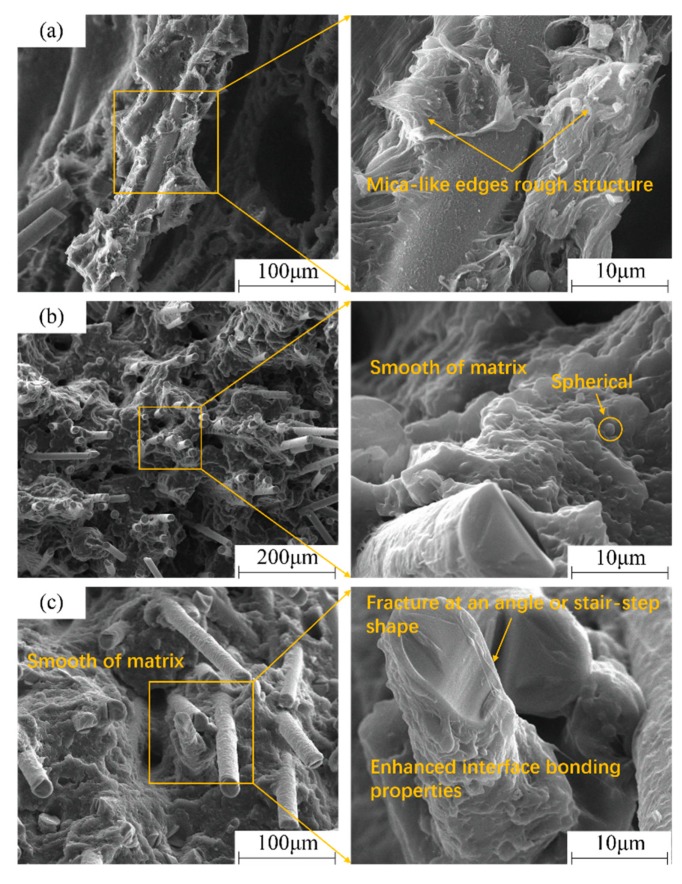
The typical fracture surface images under low, medium and high strain rates: (**a**) 0.001 s^−1^, (**b**) 1 s^−1^, and (**c**) 400 s^−1^.

The typical fracture surface images of LGFRPPs under low, medium, and high strain rates are shown in Figure 12. It can be seen that the fracture surface at low strain rate (0.001 s^−1^) has ductile failure behavior because of the high ductility of the PP matrix, as shown in Figure 12a. The matrix showed a multi-layer rough structure with mica-like edges. Figure 12b,c show the matrix surface becoming smoother at strain rates of 1 s^−1^ and 400 s^−1^, indicating a brittle fracture characteristic in comparison with low strain rate failure behavior. As the strain rates increased from 0.001 s^−1^ to 400 s^−1^, the transition from rough to smooth on the surface of the substrate indicated that the fracture behavior of the matrix changed from ductile to brittle.

A partially rough surface with a large number of voids could be observed by magnifying, as shown in Figure 12b. These voids might be relevant to the unidentified nucleating agent added during the manufacturing. The nucleating agent was added to provide a mass of nucleation sites in the solidification process [41]. Meanwhile, some white spheres began to appear due to local overheating. This was consistent with the phenomenon observed by Zrida et al. [40] in the previous study of high-speed tensile tests on a PP material. Actually, as the rate of deformation increased, the thermodynamic condition transitioned from isothermal to adiabatic. The conversion of plastic work to heat caused local adiabatic heating in the polymer matrix, but the rapid nature of impact events did not allow enough time for heat to dissipate [42,43,44]. Lienhard et al. [25,45] also found that there was a local adiabatic temperature rise in the polymer matrix at high strain rates, and the increase of the local adiabatic temperature rise would be more obvious with the higher the strain rates [42]. Therefore, the white sphere might be a droplet formed by the melting of the PP matrix due to local overheating.

By comparing the typical fracture surface images as shown in Figure 12, it was also found that the surface of the pulled-out fibers under the high strain rate was much rougher, and the PP matrix was tightly attached to the pulled-out fibers’ surface. In contrast, the fibers’ surface under the low strain rate was relatively smooth, which suggested that the interface bonding properties were improved under high strain rates conditions. It was similar to a previous study reported by Lienhard et al. [25], and it could be attributed to the higher interface fracture toughness under high strain rates level [46]. In addition, above, we explained the white spherical phenomenon. The white sphere might be a droplet formed by the melting of the PP matrix due to local overheating. When the strain rate is higher, the local adiabatic temperature-rise is more obvious. It could be seen from the white sphere that, at high strain rates, the PP matrix underwent local melting due to local adiabatic heating, which resulted in better interfacial bonding between the matrix and fibers. Simultaneously, the adiabatic temperature rise enlarged the deformation zone, impeded strain localization, and led to higher energy absorption at higher strain rates [25]. Thus, the positive strain rate sensitivity of the interface between the matrix and fiber might be caused by the interfacial bonding properties and enhanced the energy absorption capacity.

**Figure 13:**
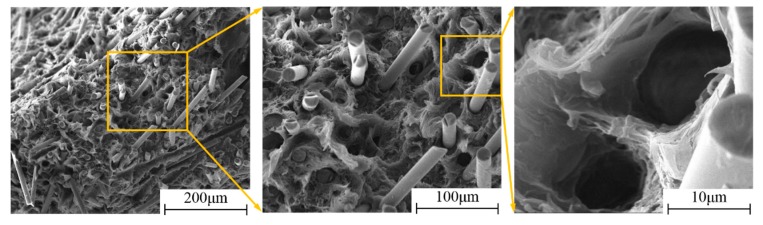
The fracture surface images at low strain rate of 0.001 s^−1^.

For static loading (low strain rate) responses, the stress-strain curves increased almost linearly at the initial stage (Figure 6), which indicated the fibers and matrix bear the load uniformly, followed by softening because of the occurrence of damage. The damage was mainly interfacial debonding and fiber pull-out. From Figure 13, a large number of irregular and indistinct holes left by the fiber pull-out was observed. At the same time, the surface of the exposed fibers was distributed with some PP matrix fragments. This meant that fiber pull-out due to interfacial debonding was the primary micro-failure mode at the low strain rate of 0.001 s^−1^.

**Figure 14:**
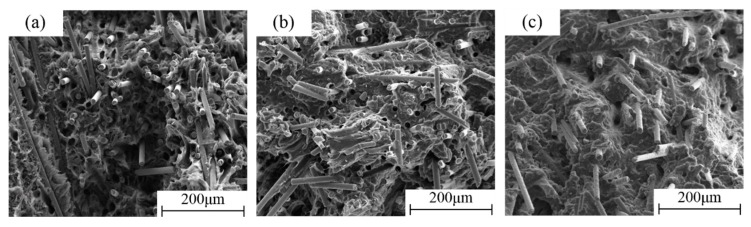
The fracture surface images under typical strain rates: (**a**) 0.001 s^−1^, (**b**) 1 s^−1^, and (**c**) 400 s^−1^.

However, the stress-strain response under the dynamic loading (medium and high strain rates) condition was fairly different. The curves tended to be non-linear in the stress increasing phase (Figure 6) due to instability of stress waves. The fracture surface images under typical strain rates of 0.001 s^−1^, 1 s^−1^, and 400 s^−1^ are shown in Figure 14. By comparing with the Figure 14a–c, it could be found that at the strain rate of 400 s^−1^, the number of fiber fractures was obviously increased, and the number of hollows left by fiber pull-out was significantly reduced. In other words, there was more fiber breakage and less fiber pull-out with the increasing strain rate. Opposite to the matrix, glass fibers showed obvious brittle fracture characteristics. The premature failure of the matrix and the improvement of the fracture toughness of the glass fiber led to the specimen further softening with the increase in the LGFRPP’s fracture strain. It was also confirmed by the fact that stress concentration effect caused a large number of fiber faults at an angle or stair-step shape under high strain rate conditions, as shown in Figure 12c.

In general, the fracture strain of LGFRPPs increased with tensile speed increasing, revealing that the fracture strain of LGFRPPs had positive strain rate sensitivity. Since the glass fiber and PP had been confirmed that they possessed positive strain rate sensitivity through tensile testing in previous studies by some scholars [39,40], the fracture strain sensitivity of LGFRPPs could be attributed to the synergistic effect caused by increased fracture strain of matrix and fiber. Additionally, the strain rate effects of the fiber and matrix interface, as mentioned earlier, also probably contributed to this behavior.

**Figure 15:**
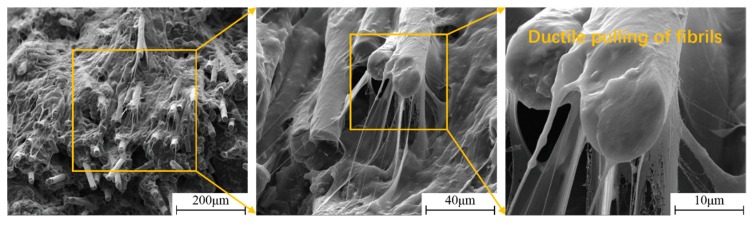
The fracture surface with ductile pulling of PP fibrils/micro-fibrils at 400 s^−1^.

In addition, the high magnification SEM micrographs showed the brittle fracture region of the LGFRPP specimens had more ductile pulling of fibrils or micro-fibrils [41] at high strain rates from Figure 15. Even though the micro-failure mode of the specimens revealed obvious brittle fracture characteristics, a large number of fibrils/micro-fibrils of the PP matrix indicated the higher fracture toughness of LGFRPPs at high strain rates, which is consistent with the tensile test results.

## 4. Conclusions

In this study, the strain rate-dependent static and dynamic mechanical behaviors and failure mechanisms of LGFRPPs were investigated. The conclusions are summarized as follows:

(1) LGFRPPs were strongly sensitive to the strain rates. As the strain rates increased from 0.001 s^−1^ to 400 s^−1^, the ultimate strength improved almost two times (from 75.4 MPa to 146.7 MPa) and the total elongation also increased nearly twice (from 2.8% to 4.6%). The elastic modulus fluctuated at 6.3 GPa, ranging from 5.2 GPa to 7.3 GPa, which remained essentially constant with ascending the strain rates.

(2) The strain near the fracture crack was larger than the average strain of the whole selected calculation area due to strain localization. The deformation mainly concentrated near the fracture crack or the location of possible failure, whereas the strain far from the fracture crack or location of possible failure hardly changed.

(3) The micro-failure modes of LGFRPPs were mainly about fibers pull-out and breakage, interfacial debonding, as well as matrix cracking. The low strain rate micro-failure mode was mainly fiber pull-out, while that of the high strain rate was mainly fiber breakage, which was caused by the improvement of the interfacial adhesion properties. In addition, the stress concentration effect caused a large number of fibers to fracture at an angle or stair-step shape under high strain rate conditions.

(4) As the increase of strain rates from 0.001 s^−1^ to 400 s^−1^, the PP matrix fracture behavior changed from ductile behavior to brittle behavior. Ductile pulling of fibrils/micro-fibrils was observed in the brittle fracture behavior of the PP matrix under high strain rate conditions, and the interfacial bonding properties were greatly improved at 400 s^−1^.

(5) The improvement of interfacial bonding properties and energy absorption capacity were fundamental reasons for the increase of the ultimate strength and fracture strain of LGFRPPs at high strain rates.

## Figures and Tables

**Table 1:** Physical and mechanical properties of fiber, PP, and composites.

Material	Density (g/cm^3^)	Tensile Strength (MPa)	Tensile Modulus (GPa)	Melt Flow Rate (g/10 min)
E-Glass fiber	2.6	2000	75	-
PP	0.9	28	1.2	2.6
LGFRPPs	1.2	73.5	5.2	-

**Table 2:** Full-field strain analysis results of the tensile process.

Strain Rate (s^−1^)	Fracture Time	Ultimate Average Strain	Fracture Strain (P_0_)
0.001	49 s	0.023	0.028
0.01	4.8 s	0.018	0.027
1	98.6 ms	0.025	0.031
10	10.7 ms	0.025	0.035
100	0.83 ms	0.027	0.035
200	0.48 ms	0.028	0.040
400	0.35 ms	0.030	0.047

**Table 3:** Tensile properties of LGFRPPs at different strain rates.

Strain Rate (s^−1^)	Young’s Modulus (GPa)	Ultimate Strength (MPa)	Total Elongation (%)
0.001	5.2	75.4	2.8
0.01	6.6	84.7	2.7
1	5.9	93.0	3.1
10	6.5	109.4	3.4
100	7.1	129.6	3.4
200	7.3	133.9	3.9
400	5.8	146.7	4.6

## References

[1] Kim D.H., Kang S.Y., Kim H.J., Kim H.S. (2019). Strain rate dependent mechanical behavior of glass fiber reinforced polypropylene composites and its effect on the performance of automotive bumper beam structure. Compos. Part B Eng..

[2] Huang C.T., Chen L.J., Chien T.Y. (2019). Investigation of the Viscoelastic Behavior Variation of Glass Mat Thermoplastics (GMT) in Compression Molding. Polymers.

[3] Zhai Z.Y., Jiang B.Y., Drummer D. (2018). Tensile Creep Behavior of Quasi-Unidirectional E-Glass Fabric Reinforced Polypropylene Composite. Polymers.

[4] Ren D.X., Chen L., Yuan Y., Li K., Xu M.Z., Liu X.B. (2018). Designing and Preparation of Fiber-Reinforced Composites with Enhanced Interface Adhesion. Polymers.

[5] Mathijsen D. (2019). Long fiber thermoplastics are a key technology in expanding existing markets for composites. Reinf. Plast..

[6] Sambale A.K., Schoneich M., Stommel M. (2017). Influence of the Processing Parameters on the Fiber-Matrix-Interphase in Short Glass Fiber-Reinforced Thermoplastics. Polymers.

[7] Wang J., Geng C., Luo F., Liu Y., Wang K., Fu Q., He B. (2011). Shear induced fiber orientation, fiber breakage and matrix molecular orientation in long glass fiber reinforced polypropylene composites. Mater. Sci. Eng. A.

[8] Henning F., Ernst H., Brüssel R. (2005). LFTs for automotive applications. Reinf. Plast..

[9] Ning H., Pillay S., Thattaiparthasarathy K.B., Vaidya U.K. (2017). Design and manufacturing of long fiber thermoplastic composite helmet insert. Compos. Struct..

[10] Balaji Thattaiparthasarathy K., Pillay S., Ning H., Vaidya U.K. (2008). Process simulation, design and manufacturing of a long fiber thermoplastic composite for mass transit application. Compos. Part A Appl. Sci. Manuf..

[11] Zhou Y., Mallick P.K. (2005). A non-linear damage model for the tensile behavior of an injection molded short E-glass fiber reinforced polyamide-6,6. Mater. Sci. Eng. A.

[12] Schoßig M., Bierögel C., Grellmann W., Mecklenburg T. (2008). Mechanical behavior of glass-fiber reinforced thermoplastic materials under high strain rates. Polym. Test..

[13] Fitoussi J., Bocquet M., Meraghni F. (2013). Effect of the matrix behavior on the damage of ethylene-propylene glass fiber reinforced composite subjected to high strain rate tension. Compos. Part B Eng..

[14] Notta-Cuvier D., Nciri M., Lauro F., Delille R., Chaari F., Robache F., Haugou G., Maalej Y. (2016). Coupled influence of strain rate and heterogeneous fibre orientation on the mechanical behaviour of short-glass-fibre reinforced polypropylene. Mech. Mater..

[15] Nciri M., Notta-Cuvier D., Lauro F., Chaari F., Maalej Y., Zouari B. (2017). Modelling and characterisation of dynamic behaviour of short-fibre-reinforced composites. Compos. Struct..

[16] Onishi P., Hashemi S. (2009). Effect of fibre concentration and strain rate on mechanical properties of single-gated and double-gated injection-moulded short glass fibre-reinforced polypropylene copolymer composites. J. Mater. Sci..

[17] Wilberforce S., Hashemi S. (2009). Effect of fibre concentration, strain rate and weldline on mechanical properties of injection-moulded short glass fibre reinforced thermoplastic polyurethane. J. Mater. Sci..

[18] Hashemi S. (2011). Temperature, strain rate and weldine effects on strength and micromechanical parameters of short glass fibre reinforced polybutylene terephthalate (PBT). Polym. Test..

[19] Okereke M.I., Paul Buckley C., Akpoyomare A.I. (2017). The mechanism of rate-dependent off-axis compression of a low fibre volume fraction thermoplastic matrix composite. Compos. Struct..

[20] Zhai Z., Jiang B., Drummer D. (2018). Strain rate-dependent mechanical behavior of quasi-unidirectional E-glass fabric reinforced polypropylene composites under off-axis tensile loading. Polym. Test..

[21] Brown K.A., Brooks R., Warrior N.A. (2010). The static and high strain rate behaviour of a commingled E-glass/polypropylene woven fabric composite. Compos. Sci. Technol..

[22] Duan S., Mo F., Yang X., Tao Y., Wu D., Peng Y. (2016). Experimental and numerical investigations of strain rate effects on mechanical properties of LGFRP composite. Compos. Part B Eng..

[23] Zhang Q., Zhang J., Wu L. (2018). Impact and energy absorption of long fiber-reinforced thermoplastic based on two-phase modeling and experiments. Int. J. Impact Eng..

[24] Bartus S.D., Vaidya U.K. (2005). Performance of long fiber reinforced thermoplastics subjected to transverse intermediate velocity blunt object impact. Compos. Struct..

[25] Lienhard J., Schulenberg L. (2018). Strain rate dependent multiaxial characterization of long fiber reinforced plastic. Compos. Part B Eng..

[26] Goel A., Chawla K.K., Vaidya U.K., Chawla N., Koopman M. (2009). Characterization of fatigue behavior of long fiber reinforced thermoplastic (LFT) composites. Mater. Charact..

[27] Phelps J.H., Abd El-Rahman A.I., Kunc V., Tucker C.L. (2013). A model for fiber length attrition in injection-molded long-fiber composites. Compos. Part A Appl. Sci. Manuf..

[28] Mehdikhani M., Aravand M., Sabuncuoglu B., Callens M.G., Lomov S.V., Gorbatikh L. (2016). Full-field strain measurements at the micro-scale in fiber-reinforced composites using digital image correlation. Compos. Struct..

[29] Jones I., Iadicola M.E. (2018). A Good Practices Guide for Digital Image Correlation. Int. Digit. Image Correl. Soc..

[30] Li L., Sun L., Dai Z., Xiong Z., Huang B., Zhang Y. (2019). Experimental investigation on mechanical properties and failure mechanisms of polymer composite-metal hybrid materials processed by direct injection-molding adhesion method. J. Mater. Process. Technol..

[31] Codolini A., Li Q.M., Wilkinson A. (2018). Mechanical characterization of thin injection-moulded polypropylene specimens under large in-plane shear deformations. Polym. Test..

[32] Röhrig C., Scheffer T., Diebels S. (2017). Mechanical characterization of a short fiber-reinforced polymer at room temperature: Experimental setups evaluated by an optical measurement system. Contin. Mech. Thermodyn..

[33] McCormick N., Lord J. (2010). Digital Image Correlation. Mater. Today.

[34] Cui J., Wang Q., Dong D., Hao J., Zhang X., Li G. (2019). A study on the constitutive equation of HC420LA steel subjected to high strain rates. J. Mater. Res..

[35] Del Rey Castillo E., Allen T., Henry R., Griffith M., Ingham J. (2019). Digital image correlation (DIC) for measurement of strains and displacements in coarse, low volume-fraction FRP composites used in civil infrastructure. Compos. Struct..

[36] Li J., Kan Q.H., Chen K.J., Liang Z.H., Kang G.Z. (2019). In Situ Observation on Rate-Dependent Strain Localization of Thermo-Induced Shape Memory Polyurethane. Polymers.

[37] Billon N., Giraudeau J., Bouvard J.L., Robert G. (2018). Mechanical Behavior-Microstructure Relationships in Injection-Molded Polyamide 66. Polymers.

[38] Szebenyi G., Hliva V. (2019). Detection of Delamination in Polymer Composites by Digital Image Correlation-Experimental Test. Polymers.

[39] Wang Y., Xia Y. (2000). Dynamic tensile properties of E-glass, Kevlar49 and polyvinyl alcohol fiber bundles. J. Mater. Sci. Lett..

[40] Zrida M., Laurent H., Grolleau V., Rio G., Khlif M., Guines D., Masmoudi N., Bradai C. (2010). High-speed tensile tests on a polypropylene material. Polym. Test..

[41] Dasari A., Misra R.D.K. (2003). On the strain rate sensitivity of high density polyethylene and polypropylenes. Mater. Sci. Eng. A.

[42] Gao Y., Xu C., He Z.-P., He Y.-L., Li L. (2015). Response Characteristics and Adiabatic Heating during High Strain Rate for TRIP Steel and DP Steel. J. Iron Steel Res. Int..

[43] Sorini C., Chattopadhyay A., Goldberg R.K. (2019). Micromechanical modeling of the effects of adiabatic heating on the high strain rate deformation of polymer matrix composites. Compos. Struct..

[44] Pan Z., Wu Z., Xiong J. (2019). High-speed infrared imaging and mesostructural analysis of localized temperature rise in damage and failure behavior of 3-D braided carbon/epoxy composite subjected to high strain-rate compression. Polym. Test..

[45] Lienhard J., Böhme W. (2015). Characterisation of resin transfer moulded composite laminates under high rate tension, compression and shear loading. Eng. Fract. Mech..

[46] Zhai Z., Gröschel C., Drummer D. (2016). Tensile behavior of quasi-unidirectional glass fiber/polypropylene composites at room and elevated temperatures. Polym. Test..

